# Effect of Zinc on *Microcystis aeruginosa* UTEX LB 2385 and Its Toxin Production

**DOI:** 10.3390/toxins12020092

**Published:** 2020-01-30

**Authors:** Jose L. Perez, Tinchun Chu

**Affiliations:** Department of Biological Sciences, Seton Hall University, South Orange, NJ 07079, USA

**Keywords:** cyanobacteria, cyanotoxins, microcystin, metal, zinc, *Microcystis aeruginosa*

## Abstract

Cyanobacteria harmful algal blooms (CHABs) are primarily caused by man-made eutrophication and increasing climate-change conditions. The presence of heavy metal runoff in affected water systems may result in CHABs alteration to their ecological interactions. Certain CHABs produce by-products, such as microcystin (MC) cyanotoxins, that have detrimentally affected humans through contact via recreation activities within implicated water bodies, directly drinking contaminated water, ingesting biomagnified cyanotoxins in seafood, and/or contact through miscellaneous water treatment. Metallothionein (MT) is a small, metal-sequestration cysteine rich protein often upregulated within the stress response mechanism. This study focused on zinc metal resistance and stress response in a toxigenic cyanobacterium, *Microcystis aeruginosa* UTEX LB 2385, by monitoring cells with (0, 0.1, 0.25, and 0.5 mg/L) ZnCl_2_ treatment. Flow cytometry and phase contrast microscopy were used to evaluate physiological responses in cultures. Molecular assays and an immunosorbent assay were used to characterize the expression of MT and MC under zinc stress. The results showed that the half maximal inhibitory concentration (IC_50_) was 0.25 mg/L ZnCl_2_. Flow cytometry and phase contrast microscopy showed morphological changes occurred in cultures exposed to 0.25 and 0.5 mg/L ZnCl_2_. Quantitative PCR (qPCR) analysis of selected cDNA samples showed significant upregulation of *Mmt* through all time points, significant upregulation of *mcyC* at a later time point. ELISA MC-LR analysis showed extracellular MC-LR (µg/L) and intracellular MC-LR (µg/cell) quota measurements persisted through 15 days, although 0.25 mg/L ZnCl_2_ treatment produced half the normal cell biomass and 0.5 mg/L treatment largely inhibited growth. The 0.25 and 0.5 mg/L ZnCl_2_ treated cells demonstrated a ~40% and 33% increase of extracellular MC-LR(µg/L) equivalents, respectively, as early as Day 5 compared to control cells. The 0.5 mg/L ZnCl_2_ treated cells showed higher total MC-LR (µg/cell) quota yield by Day 8 than both 0 mg/L ZnCl_2_ control cells and 0.1 mg/L ZnCl_2_ treated cells, indicating release of MCs upon cell lysis. This study showed this *Microcystis aeruginosa* strain is able to survive in 0.25 mg/L ZnCl_2_ concentration. Certain morphological zinc stress responses and the upregulation of *mt* and *mcy* genes, as well as periodical increased extracellular MC-LR concentration with ZnCl_2_ treatment were observed.

## 1. Introduction

Cyanobacteria harmful algal blooms (CHABs) are described as toxigenic or irritating biomasses of mostly oxygenic, photosynthetic bacteria on the rise worldwide due to anthropogenic eutrophication (via excessive P and N loading) and increasing climate-change conditions [1,2,3,4,5,6]. Oftentimes, metal pollutant runoff in water systems may also affect the ecological interaction of a given CHAB population [7]. Certain essential element processes, such as iron’s regulation by (FUR) uptake regulators, may act as growth-limiting factors in established cyanobacteria populations [1,8]. Additionally, the effects of different heavy metal compounds and concentrations on varying cyanobacteria populations and cyanotoxin production have been demonstrated in a few studies [9,10,11].

The rising occurrence of global CHABs may lead to a greater probability of human exposure and animal detrimental effects through environmental interactions [12,13]. Generally, humans may be exposed to CHABs cyanotoxins or irritants via recreation activities in contaminated water sources, imbibement or infusion (via medical dialysis) of contaminated water, ingestion of biomagnified cyanotoxins through food sources, and possible long-term chronic exposure through ingestion [14,15,16]. Cyanotoxins may structurally present as alkaloids, polyketides, cyclic peptides, and amino acid complexes, and are potently classified as neurotoxins, hepatotoxins, or cytotoxins [17,18]. The toxic health effects of cyanotoxins in humans are both varied and dependent on the class, but effects may display from mild symptoms to severe and fatal [16,19]. 

Aside from escalating incidences of detrimental effects to organisms, CHABs will most likely play an increasing role in global economic dynamics. The greater occurrence of global CHABs have reported some monitoring and contingency plan estimate costs of 10,000 s–1,000,000 s USD per country per year [20]. While these numbers do not often reflect loss of recreation revenue, cost of treatment actions, or socio-economic strategies (for anthropogenic P-N load reduction), an estimate report that took these components into account (within the United States) calculated potential losses in the billions USD per year [21].

*Microcystis* spp. and the hepatotoxins microcystins (MCs), have been identified as several of the most commonly encountered freshwater CHABs species and cyanotoxins, respectively, on a global scale and within the U.S. [22,23]. As such, studies evaluating both *Microcystis* and MCs have increased in importance in the recent years. Adding to the gravity of these considerations, examples of toxigenic *Microcystis* outcompeting non-toxigenic strains within elevated surface water temperature parameters have been observed [24,25]. This indicates a potentially competitive selection of toxigenic strains over non-toxigenic strains under climate change conditions. *Microcystis aeruginosa* is a commonly identified species of *Microcystis* within many CHABs, and have been found on all continents except for the Antarctic [26]. *M. aeruginosa* are typically 2–8 µm wide, unicellular planktonic cyanobacteria that possess variable, intracellular gas vesicles and often form colonies in natural and eutrophic conditions [27,28,29]. MCs (~995 Da for MC-LR) are water soluble, monocyclic heptapeptides that can be produced by freshwater, terrestrial, and benthic cyanobacteria. The most common genus and species producing a given variant(s) of the ~ 200 identified MCs are: *Microcystis, Chroococcus, Planktothrix, Anabaena, Nostoc, Oscillatoria, Hapalosiphon,* and *Phormidium* [18,30,31]. MCs are biosynthesized by a non-ribosomal peptide synthetase/polyketide synthase complex (known as microcystin synthetase) [18], and vary in toxicity depending on the L-amino acid components and binding capacity to receptor sites [32]. The role of MCs in the extracellular environment remains largely undetermined, but several studies have proposed an intercell signal-like characteristic where environmental conditions and introduced MCs enhanced *mcy* gene and toxin production in established cell populations [33,34]. Therefore, further evaluation of stimulatory MC release to the extracellular environment and their possible extracellular functions with environmental factors is of great importance. Zinc (Zn) is an important mineral integral to the physiological functions of all organisms. Zn naturally occurs as a trace element in world average river waters (0.27–27 µg/L), as ionic complexes in continental crust and world soil averages (70 mg/kg, respectively), and as precipitating minerals, organic, and inorganic compounds in water (~3.25 µg/L in worldwide, clean drinking water), but organic and inorganic zinc compounds are oftentimes identified as metal runoff contaminants within aqueous environments [35,36,37]. A comparison of the average Zn levels originating from a continental crustal average (52 ppm) was found to be significantly different to an anthropogenic impacted region, New York Harbor, United States (188–244 ppm), showing a further trend in heavy metal accumulation within water environments proximal to urbanized and industrial sources [38,39]. The toxicity of ZnCl_2_ has been known in many organisms [37,40,41] and can cause external irritation, severe inflammation, and gastrointestinal toxicity dependent on percent ingestion [42]. Cyanobacteria sensitivity, resistance or adaptive sequestration of heavy metal concentrations has been documented within both colonial CHABs and unicellular species [7,43,44,45]. Aside from the observed zinc metal-complexing potential of MCs (MC-LR-Zn = −617 ± 7 kcal mol^−1^; MC-RR-Zn =−777 ± 9 kcal mol^−1^) [46], metallothioneins (MTs) are well documented metal-cation chelating, cysteine-rich proteins (<10 kDa) ubiquitously found in prokaryotes and eukaryotes [47,48]. MTs have been shown to be upregulated in different species of cyanobacteria when exposed to Zn^2+^ or Cd^2+^ concentrations while remaining relatively constant at basal levels [49,50]. Because of the variability of the MC synthetase gene cluster (encoding *mcyABC*–*mcyD–J*) in different cyanobacteria clades and within strains [51] and a relative conservation of MT cysteine domain sequences and motifs across cyanobacteria and bacteria [48], these genes may be possible quantification method candidates involving heavy metal zinc response and resistance in identified MC producing *Microcystis* species and strains.

While the use of quantitative PCR (qPCR) has yielded both successes and noncorrelation in relating MC synthetase gene copy numbers or gene expressions with collection site MC concentrations [52], qPCR remains a very powerful and accessible technique for the study of gene regulation in known toxigenic or identified cyanobacteria species [53]. Along with other quantitative analysis (HPLC, LC/MS, ELISA) and sequencing profiles, it may lead to the development of known metal-response gene standards for important identified toxic cyanobacteria species and strains. These parameters may better assist in determining toxic vs. non-toxic cyanobacteria response and resistance to heavy metal pollution. 

The aim of this study was (1) to study the growth and physiological effects of zinc concentrations on an established toxigenic *M. aeruginosa* strain; (2) to design *mcyC, mcyE,* and *Mmt* qPCR oligonucleotides to quantify *mcyC, mcyE* and *Mmt* relative gene expression profiles of this strain treated with varying Zn^2+^ concentrations; and (3) to determine relative quantitation of MC-LR equivalents within ZnCl_2_-treated *M. aeruginosa* using intracellular and extracellular portions. 

## 2. Results

### 2.1. Growth Response to ZnCl_2_ in M. Aeruginosa UTEX LB 2385 

**Figure 1:**
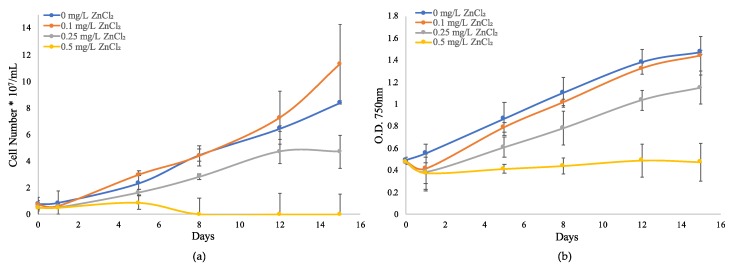
Growth curves of *Microcystis aeruginosa* UTEX LB 2385 treated with 0, 0.1, 0.25, and 0.5 mg/L ZnCl_2_. (**a**) Direct count of cell number was made via hemocytometer through 15 days. (**b**) Turbidity was evaluated via optical density (OD_750 nm_) readings through 15 days. Data is presented as mean ± SD of three replicates.

To better evaluate the zinc metal resistance and response mechanisms of a globally important toxigenic cyanobacteria species, *M. aeruginosa* UTEX LB 2385 cultures were used for the observation of physiological responses to long-term ZnCl_2_ concentrations exposure via growth monitoring (Figure 1). For *M. aeruginosa*—UTEX LB 2385 cells exposed to 0.1 mg/L ZnCl_2_, the cell concentration was similar to 0 mg/L ZnCl_2_ (control) through 15 days (Figure 1). However, the average turbidity for *M. aeruginosa* UTEX LB 2385 culture cells exposed to all ZnCl_2_ concentrations decreased below the initial control measurement (optical density, OD_750 nm_ ≈ 0.47) for 0.1, 0.25, and 0.5 mg/L ZnCl_2_ by Day 1 (Figure 1b). When compared to control cells, *M. aeruginosa* UTEX LB 2385 culture cell numbers exposed to 0.25 mg/L ZnCl_2_ were reduced by ~28% by Day 15 ((4.73 ± 1.243/8.41 ± 0.122) × 100) (Figure 1a). At the highest concentration treatment (0.5 mg/L ZnCl_2_), *M. aeruginosa* UTEX LB 2385 culture cells were almost completely inhibited in comparison to 0.25 mg/L ZnCl_2_ cells by the end of the growth monitoring period. This concentration (0.5 mg/L ZnCl_2_) may therefore present as the ZnCl_2_ minimum inhibitory concentration (MIC) for *M. aeruginosa* UTEX LB 2385 within this study (Figure 1). Aside from these observations, *M. aeruginosa* UTEX LB 2385 culture cells treated with 0.25 and 0.5 mg/L ZnCl_2_ were observed via hemocytometer to possess larger aggregate cell clusters and extracellular debris compared to control cells. 

### 2.2. Phase Contrast Microscopy Imaging in ZnCl_2_ Exposed Cells

**Figure 2:**
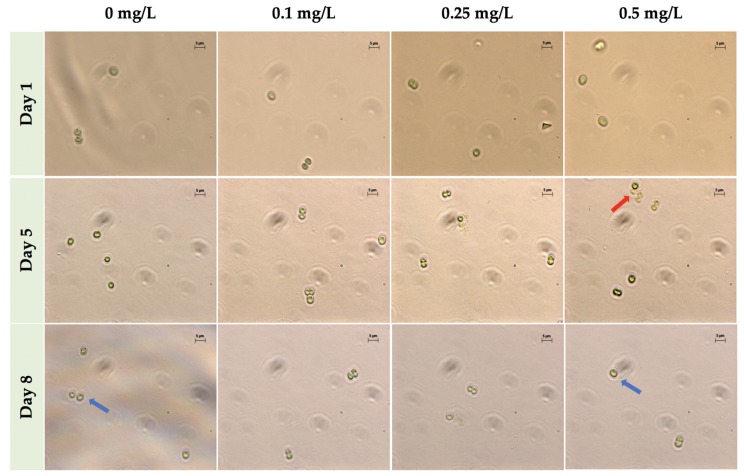
Phase-contrast microscopy of *Microcystis aeruginosa* UTEX LB 2385 treated with 0, 0.1, 0.25, and 0.5 mg/L ZnCl_2_ at Days 1, 5, and 8, 1000× total magnification. Although greatly reduced in cell number, *M. aeruginosa* UTEX LB 2385 cells treated with 0.5 mg/L ZnCl_2_ presented morphology similar to 0 mg/L cells through Day 8, as indicated by blue arrows. The red arrow indicates a small cluster of multi-cell aggregation at Day 5 for 0.5 mg/L ZnCl_2_ cells. Scale bar: 5 μm.

*M. aeruginosa* UTEX LB 2385 culture cells exposed to 0.25 mg/L ZnCl_2_ possessed morphological and size characteristics similar to the control cells through eight days as single cells (Figure 2), but also showed an increase in multi-paired cell aggregation and size by Days 5 and 8 when observed via phase contrast microscopy. Additionally, though 0.5 mg/L ZnCl_2_ treatment greatly inhibited *M. aeruginosa* UTEX LB 2385 culture cell numbers by Day 8 compared to the control, there was an observable number of cells possessing similar *M. aeruginosa* UTEX LB 2385 morphology to control cells (Figure 2). It also appeared a greater observable amount of multi-cell aggregation within 0.5 mg/L ZnCl_2_ treated cells versus control cells. Flow cytometry was used to further evaluate the population sizes of ZnCl_2_ treated *M. aeruginosa* UTEX LB 2385.

### 2.3. Flow Cytometry 

**Figure 3:**
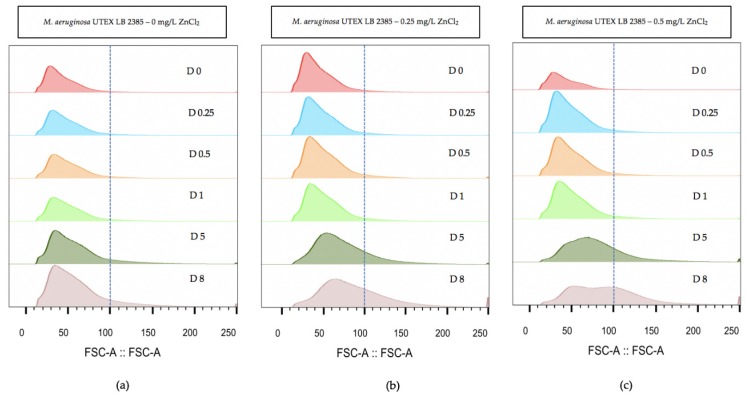
Flow cytometry analysis of population size (forward scatter: FSC-A) histograms within *Microcystis aeruginosa* UTEX LB 2385 treated with 0, 0.25, and 0.5 mg/L ZnCl_2_. (**a**) 0 mg/L ZnCl_2_ cells showed relative uniform FSC-A size through eight days. (**b**) 0.25 mg/L ZnCl_2_ cells and (**c**) 0.5 mg/L ZnCl_2_ cells showed increased FSC-A population size at Day 8 compared to control cells. Histograms were generated via FlowJo software analysis. (D represent days).

Flow cytometry was used over the eight day time-course to evaluate the relative morphology size of *M. aeruginosa* UTEX LB 2385 culture cell populations treated with the highest ZnCl_2_ concentrations (0.25 and 0.5 mg/L). The flow cytometry measurement (FCM) histogram profiles were ungated for subpopulations to evaluate overall distribution of single cell + multi-cell aggregates. Figure 3 demonstrates a histogram profile of 0, 0.25, and 0.5 mg/L ZnCl_2_ treated *M. aeruginosa* UTEX LB 2385 cultures through size (forward scatter: FSC-A) parameter. By Day 8, (Figure 3) the FSC-A histograms of *M. aeruginosa* UTEX LB 2385 cultures treated with 0.25 and 0.5 mg/L ZnCl_2_ had positively shifted in comparison to the control cultures, with increasing FSC-A measurement (Figure 3). *M. aeruginosa* UTEX LB 2385 cultures treated with 0.5 mg/L ZnCl_2_ showed a distinct, larger population size and two small peaks at Day 8 compared to the control—supporting both hemocytometer cell count and microscopic observations of greater numbers of multi-cell aggregates (Figure 3c(D8)). FlowJo software statistical analysis showed average 0.5 mg/L ZnCl_2_ treated cells FSC-A measurement was approximately 91 versus 62 for 0 mg/L ZnCl_2_ treated cells at Day 8. 

### 2.4. Quantitative Polymerase Chain Reaction (qPCR) Analysis of M. Aeruginosa UTEX LB 2385 

**Figure 4:**
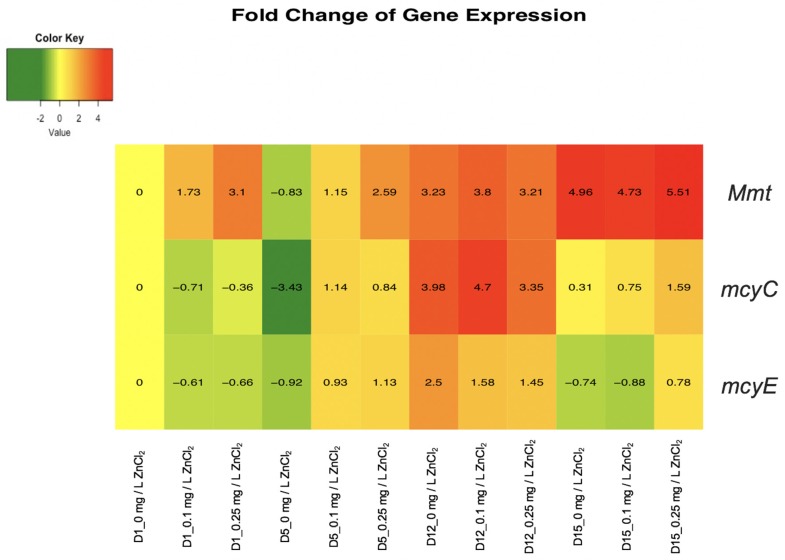
qPCR analysis after cDNA synthesis, of three genes: *Mmt*, *mcyC*, and *mcyE* as potential zinc metal response genes in *M. aeruginosa* UTEX LB 2385. *Mmt* (top row) overall expression was shown to be highest in 0.25 mg/L ZnCl_2_ treated cells, with significant (Day 1) upregulation in comparison to 0 and 0.1 mg/L ZnCl_2_ treatment. *mcyC* and *mcyE* (middle and bottom rows, respectively) were distinct in expression patterns, with *mcyC* gene significantly upregulating at Day 12, showing a fold-increase >2.5 in ZnCl_2_ treated cells. For 0.1 and 0.25 mg/L ZnCl_2_ treated cells, Day 12 showed gene expression increase for *Mmt* and *mcyC* genes. (D represent days; 0, 0.1, 0.25 mg/L represent ZnCl_2_ concentrations; color scale of yellow refers to no change, green-downregulation, orange-red-upregulation).

The qPCR analysis results were calculated as fold change gene expressions of *Mmt*, *mcyC*, and *mcyE* relative to the *M. aeruginosa* UTEX LB 2385 16S ribosomal RNA internal control, within the specific time period and samples (Days 1, 5, 12, 15 for 0, 0.1, and 0.25 mg/L ZnCl_2_) and the initial 0 mg/L ZnCl_2_ Day 1 sample. Fold change gene expression values were expressed as [2^−ΔΔCt^] and evaluated as a combined [log_2_(RQ)] heatmap within RStudio statistical software. The genes expressed in log2 > 0 were represented within an orange-red color scale and indicate upregulation as a response to ZnCl_2_ treatment, and the genes in log2 < 0 were represented within a green color scale and indicated downregulation as a response to ZnCl_2_ treatment. Welch two sample *t*-tests of 16S rRNA endogenous average C_t_ values control were found to not be statistically different within each sample condition (all *p* values > 0.05). The specific sample treatment and days were abbreviated as D1_0 mg/L ZnCl_2_, D1_0.1 mg/L ZnCl_2_, D1_0.25 mg/L ZnCl_2_, etc. Correlating to Day 1 treatments for 0, 0.1, and 0.25 mg/L ZnCl_2_, respectively (Figure 4). 

*M. aeruginosa* UTEX LB 2385 *Mmt* expression displayed an overall upregulation over the time course period of 15 days, with 0.25 mg/L ZnCl_2_ treated cells showing > 3-fold expression by Day 1 (Figure 4). By Day 15 there was a > 5-fold *Mmt* gene expression within 0.25 mg/L ZnCl_2_ treated cells, demonstrating a steady increase of *Mmt* gene expression within this study. Additionally, the overall trend throughout 15 days showed 0.1 and 0.25 mg/L ZnCl_2_ treated cells to be comparable or greater than control (0 mg/L ZnCl_2_) cells (Figure 4—top row).

*M. aeruginosa* UTEX LB 2385 *mcyE* gene expression showed the least change of expression between both *mcy* genes profiles, with an initial slight *mcyE* down-regulation by Day 1 in 0.1 and 0.25 mg/L ZnCl_2_ treated cells. For 0.25 mg/L ZnCl_2_ treated cells, *mcyE* gene expression showed slight up-regulation from Days 5 to 15 in comparison to the control cells (Figure 4—bottom row). The expression of *mcyC* was shown to be significantly upregulated at Day 12, with >4.5 fold-increase in 0.1 mg/L ZnCl_2_ treated cells and >3 fold-increase in 0.25 mg/L ZnCl_2_ treated cells (Figure 4—middle row). The 0.25 mg/L ZnCl_2_ treated cells also showed a slight *mcyC* upregulation in comparison to both the control and 0.1 mg/L ZnCl_2_ treated cells at Day 15 (Figure 4—middle row, right). The *mcyC* gene expression profile in ZnCl_2_ treated cells showed an up-regulation trend at Days 5 and 12 in comparison to all other time periods (Figure 4—middle and bottom rows, center). 

### 2.5. ELISA Analysis

ELISA quantitative analysis of 0, 0.1, 0.25, and 0.5 mg/L ZnCl_2_ treated *M. aeruginosa* UTEX LB 2385 cells was performed as MC-LR equivalents using an MC-LR standard curve generated with MC-LR standards (0–5 µg/L). A standard 2^nd^-order polynomial equation (y = 7.5536x^2^ − 54.927x + 96.06) was used to calculate MC-LR equivalents below the 2.5 µg/L standard range. The ELISA analysis was performed on extracted MC-LR intracellular and extracellular samples at time periods (Day: 1, 5, 8, 12, and 15). Total MC-LR (µg/cell) quota was calculated by adding intracellular and extracellular MC-LR equivalents and dividing by average cells/L. The Pearson’s *r* correlation showed that total MC-LR (µg/cell) quota was positively correlated with ZnCl_2_ concentration (*p* < 0.05, *r* = 0.6).

**Figure 5:**
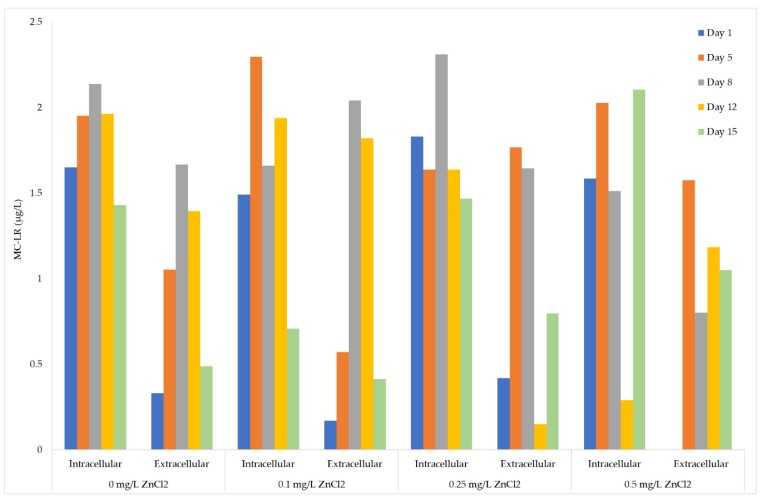
ELISA quantitative analysis of intracellular and extracellular MC-LR (µg/L) equivalents within *Microcystis aeruginosa* UTEX LB 2385 treated with 0, 0.1, 0.25, and 0.5 mg/L ZnCl_2_ at Days: One, 5, 8, 12, 15. Extracellular MC-LR equivalents (µg/L) of 0.1 mg/L ZnCl_2_ cells showed a 18% and 23% increase for Days 8 and 12 compared to control; 0.25 and 0.5 mg/L ZnCl_2_ treated cells showed a ~40% and 33% increase of extracellular MC-LR, respectively, as early as day 5. The 0.25 mg/L ZnCl_2_ extracellular MC-LR (µg/L) equivalents decreased and increased from Days 8 to 15, and 0.5 mg/L ZnCl_2_ intracellular MC-LR (µg/L) intracellular measurements decreased and increased from this same time point. All samples were performed as duplicate replicates.

The data was presented as intracellular-extracellular MC-LR (µg/L) equivalents to better evaluate relative concentrations of MC-LR within each sample portion. The extracellular MC-LR (µg/L) equivalents for Days 8 and 12 of *M. aeruginosa* UTEX LB 2385 cultures treated with 0.1 mg/L ZnCl_2_ were comparable to 0 mg/L ZnCl_2_ control cells (18% increase of MC-LR equivalents for Day 8: {(1.67/2.04) × 100}; 23% increase of MC-LR equivalents for Day 12: {(1.40/1.82) × 100}) (Figure 5). Day 12 extracellular MC-LR (µg/L) equivalent measurements for 0.25 mg/L ZnCl_2_ treated cells decreased from Day 8, but once again increased comparable to control cells at Day 15 (Figure 5). This same time period saw a 40% increase in cell number/mL biomass within 0.25 mg/L ZnCl_2_ treated cells (see Figure 1a above) and a greater amount of multicell aggregates at Day 8. The 0.25 and 0.5 mg/L ZnCl_2_ treated cells possessed higher extracellular MC-LR (µg/L) equivalents at Day 5 compared to control cells {~ 40% increase for 0.25 mg/L ZnCl_2_ cells (1.05/1.77) × 100; ~ 30% increase for 0.5 mg/L ZnCl_2_ cells (1.05/1.57) × 100) (Figure 5). From Days 8 through 15 of this study, 0.5 mg/L ZnCl_2_ treated cells yielded significantly higher total MC-LR (µg/cell) quota yield (student’s t-test *p* < 0.05) than all other ZnCl_2_ treatment. However, the cell concentration had decreased from Day 5 to Day 8 for 0.5 mg/L ZnCl_2_ treated cells as stated above. This would indicate possible release of MCs into the extracellular matrix from lysed cells rather than increased MC production due to ZnCl_2_ concentration.

## 3. Discussion

Zn is a classified essential, transitional metal ubiquitously involved in numerous cellular biochemical pathways, and is often found within enzyme cavities and protein infrastructures. Zn is often employed in galvanization processes, vulcanization procedures, automobile applications, coating alloy formulations, and various manufacturing components where it often enters the environment in a sequential fashion after ZnO is formed [54]. Environmental chloride ions and pH often form ZnCl_2_ near man-made sources high in processed Zn [55].

The interactions of zinc and heavy metal pollution with organisms in naturally occurring and man-made aquatic environments presents its own immediate and long-term problems in our increasingly industrial societies. As an example, a surface river, estuary, and bay sediment study in highly industrial Jinzhou Bay, China found Cu, Zn, Pb, and Cd heavy metals concentrations to be correlated to man-made industrial activities, and has been identified as a polluted ecological risk [56]. Relatively, documented metal resistance and stress response mechanisms within cyanobacteria populations necessitates that focus shifts towards risk assessment, prevention, and treatment. *Synechococcus* sp. IU 625 cultures were shown to be highly tolerant to 25 mg/L ZnCl_2_ treatment, and a similar strain showed comparable growth to control when treated with 0.1 mg/L and 0.5 mg/L HgCl_2_ [43,50]. Relatively and conversely, a total-protein profile of an *Anabaena flos-aquae* isolate showed progressive decrease upon treatment with increasing concentrations of CdCl_2_ and CuCl_2_—with the highest concentrations presenting the most considerable biophysiological and biomass damage [57].

CHABs-associated species and strains often found in metal polluted environments must be given special attention due to their potential survivability and resultant detrimental effects (i.e., cyanotoxin release). Although distinct environmental factors have been observed to contribute to favorable conditions conducive to cyanobacteria growth, the specific combinations of effects that causes heavy metal tolerance or resistance in cyanobacteria population are not well understood, or in fact, remain unknown. 

Our results showed that *M. aeruginosa* UTEX LB 2385 is tolerant to ZnCl_2_ concentrations of 0.1 and 0.25 mg/L, with 0.1 mg/L ZnCl_2_ treated cell numbers and turbidity measurements being similar to control cells through 15 days (Figure 1a,b). All cell concentrations were found to be statistically similar through five days (student’s t-test *p* > 0.05 for 0 mg/L and 0.5 mg/L ZnCl_2_ treated cells/mL). Further, 0.5 mg/L ZnCl_2_ concentration was found to largely inhibit cell concentration (cell/mL) by Day 15 {(0.01/8.41) × 100 ~ 99.9% inhibition} and decreased the turbidity of the culture. This result is similar to gradual cell density decrease with increasing Zn^2+^ concentration [11]. Additionally, it was also similar to *M. aeruginosa* Kütz 854 cultures showing chlorophyll and phycobiliprotein decrease at CdCl_2_ concentrations of 1 and 2 µM, and a bactericidal reaction at 4 µM CdCl_2_ treatment [58]. Both a toxic *M. aeruginosa* (FACHB-905) and a non-toxic strain (FACHB-469) were shown to accumulate Zn^2+^ and Cd^2+^ intracellularly as an uptake metal concentration vs. four-hour time rate [59]. This previous study observed intracellular Zn concentration was a good predictor of Zn toxicity in *M. aeruginosa*. In a different study, a 10 nM treatment of Zn as a free ion concentration did not decrease microcystin/chlorophyll-*a* (μg/μg) in comparison to UVR and Cu^2+^ metal treatments of *M. aeruginosa* strains (UTEX LB 2385 and LE3), but total added Zn to lake water did affect the final biomass and growth rates [60]. Different *M. aeruginosa* chlorophyll concentrations were also found to be more stable to CdNO_3_ treatment than to Pb(NO_3_)_2_, and only showed chlorophyll decrease at 20 mg/L Cd after 24 h [61]. Additionally, low levels of both Cd and Pb (1–5 mg/L) resulted in increases of chlorophyll fluorescence during 24-h incubation, and showed that specific *M. aeruginosa* strain was not inhibited at those concentrations. Conversely, Fe^2+^ and Fe^3+^ was shown to increase *M. aeruginosa* cell density with increasing concentrations up to 12 mg/L [11]. Lastly, *M. aeruginosa* (FACHB-905) cultures were observed to show levels of resistance and recovery to treatment with arsenic (III) concentrations (0.01, 0.1, and 1 mg/L) after 48 h incubation, and only showed marked damaging effects to growth and carotenoids production at 10 mg/L [62]. These observations indicate that heavy metal inhibition, possible resistance, or resultant growth rate in *M. aeruginosa* is likely dependent on strain and metal species.

Cell counts, phase-contrast microscopy, and flow cytometry (FSC-A) histogram profile showed that *M. aeruginosa* UTEX LB 2385 treated with 0.25 and 0.5 mg/L ZnCl_2_ possessed larger amounts of multi-cell aggregates compared to 0 and 0.1 mg/L ZnCl_2_ treated cells at Day 8 (Figure 3c(D8)). Progressive positive shifting of histograms occurred from Day 5 to Day 8 referenced from FCM of (100) FSC-A for 0.25 and 0.1 mg/L ZnCl_2_ treated cells (Figure 3). Future experiments should concentrate on the dynamics of aggregation formation as it relates to intracellular molecular profile, in response to increasing ZnCl_2_ concentrations. Aggregation of many *Microcystis* species is thought to contribute to possible natural bloom formation, and subsequent protection from environmental factors such as heavy metals [63]. *M. aeruginosa* were shown able to survive in low concentrations of variable heavy metal compounds, and *Microcystis* blooms showed a capacity to bioaccumulate and sequester heavy metal compounds within historically eutrophic lakes [7,10,58].

qPCR analysis of 0.1 and 0.25 mg/L ZnCl_2_ treated *M. aeruginosa* UTEX LB 2385 showed that *Mmt* gene expression within this strain was significantly upregulated by ZnCl_2_ concentration dependent and time period dependent parameters (Figure 4—top row). MTs are well documented divalent/monovalent metal sequestrating, intracellular proteins that are ubiquitously found from prokaryotes to humans. Our results are supported by the observations of cyanobacteria *Synechococcus* sp. IU 625, which showed a marked upregulation of *smtA* at 25 mg/L ZnCl_2_ when analyzed via transcriptome analysis and qPCR analysis [50], and a relative, *Synechococcus elongatus* PCC 7942, which showed upregulation of *smtA* with Zn treatment [64]. The expression of *mcyC* was significantly upregulated at Day 12 for 0.1 and 0.25 mg/L ZnCl_2_ treatment, while *mcyE* only showed slight upregulation for 0.1 and 0.25 mg/L ZnCl_2_ treatment cells at this same time point (Figure 4—middle and bottom row). While there is an obvious correlation of *mcyC* expression levels and ZnCl_2_ treatment within this study, *mcyE* expression level trends varied by both concentration and time period (0 and 0.1 mg/L ZnCl_2_ showed slight downregulation at Day 15 vs. *mcyC*) and was not predictive of ZnCl_2_ concentration response (Figure 4—middle and bottom row, right). Additional *mcy* gene expression, metal treatment studies should be performed to better assess possible intracellular MC functions. Though *mcyC* function in *Microcystis aeruginosa* PCC 7806 has been evaluated to be important in the final condensation reaction release of the entire MC heptapeptide [65], further analysis must be performed to evaluate *mcyC* gene relation to heavy metal stress response, MC-variant identification, and total MC release to the extracellular environment. The intracellular roles of MCs are still largely undetermined, but a possible siderophore-like function towards zinc and iron may exist under specific intracellular conditions [66]. Molecular modeling for MC-LR metal binding showed that the total potential energy (Kcal⋅mol^−1^) (relative stability) for MC-LR was: Zn > Cu ≥ Fe ≥ Mg > Ca [46].

ELISA quantitative analysis showed MC-LR (µg/L) were present in all ZnCl_2_ treated intracellular and extracellular *M. aeruginosa* UTEX LB 2385 culture portions (Figure 5). There was a higher correlation of MC-LR (µg/cell) yield to 0.5 mg/L ZnCl_2_ treatment cells from Day One to Day 15 versus 0, 0.1, and 0.2 mg/L ZnCl_2_ treated cells (*p* < 0.05, *r* = 0.6). This correlation was inversely associated with the decreased cell biomass through 15 days, indicating release of MC-LR into the extracellular environment. It was observed that intracellular microcystin production was decreased with increasing multiple metal concentrations, indicating a possible distribution of microcystins (intracellularly to extracellularly) due to cell lysis and/or cellular damage [10]. The 0.25 and 0.5 mg/L ZnCl_2_ treated cells showed a ~40% and 33% increase of extracellular MC-LR equivalents, respectively, as early as Day 5 (Figure 5). Although 0.5 mg/L ZnCl_2_ cell concentration was largely inhibited by the end of the monitor period (99.9% inhibition), a number of multi-cell aggregates and a very low cell number of individual cells (1 × 10^5^ cells/mL) were present at Day 15. The sum of all measured extracellular and intracellular MC-LR (µg/L) concentration through 15 days was slightly higher for cultures treated with 0, 0.1, and 0.25 mg/L ZnCl_2_ than for the 0.5 mg/L ZnCl_2_ treated culture. These results are supported by observations that total MC presence is largely associated with overall cell biomass [67]. Extracellular MC-LR (µg/L) equivalent measurements for 0.25 mg/L ZnCl_2_ treated cells fluctuated between Days 8, 12, and 15 (1.64, 0.15, 0.80 µg/L, respectively), but was decreased from Day 5 (Figure 5). Interestingly, the presence of observed multi-cell aggregates in both 0.25 and 0.5 mg/L ZnCl_2_ treated cells from Days 8 through 15 coincided with this observation. These structures may have possibly affected MC interactions with higher ZnCl_2_ concentrations (0.25 and 0.5 mg/L ZnCl_2_) in the extracellular environment for the measured time period. A further study of multi-cell aggregation, Exopolysaccharides (EPS) production, and the MC role in the extracellular environment must be evaluated to determine interaction dynamics and possible association. These observations are supported by the following studies. The application of 0.5 mg⋅L ^−1^ Zn^2+^ was shown to increase the dissolve organic carbon (DOC) concentration of *M. aeruginosa* (FACHB 469) into the surrounding media by a 18-day study [68]. Furthermore, it was shown that the addition of MC-RR (0.25–10 µg⋅L^−1^) significantly enlarged *Microcystis* colony size, specifically increasing EPS production [69]. There are a number of undetermined factors concerning the interaction of MCs-metal species in the extracellular environment, and these factors are complicated by the presence of EPS, amino acids, and miscellaneous molecules produced by stress-responsive cells. 

Further associative evaluation of *Microcystis* aggregation and the associated multi-cell components to heavy metal stress is necessary to determine potential resistance mechanisms and survivability dynamics of this cyanobacteria. A future transcriptomic evaluation of Zn heavy metal response in *M. aeruginosa* is necessary to profile which intracellular and extracellular localizing genes are in regulation, and to distinguish this important CHAB-associated cyanobacteria species from other cyanobacteria species. Finally, the role of MCs in the extracellular environment must be further determined to understand cyanobacteria-cyanobacteria environment dynamics.

## 4. Conclusions

This study exhibits the ZnCl_2_ metal stress response and resistance capabilities that a universal CHAB species, *Microcystis aeruginosa* (in this study with strain *M. aeruginosa* UTEX LB 2385) possesses, and the potential survivability this species demonstrates in increasing polluted aquatic environments. *M. aeruginosa* UTEX LB 2385 was observed to survive ZnCl_2_ concentrations of up to 0.25 mg/L, with increasing biomass through 15 days. Though mostly inhibited, 0.5 mg/L ZnCl_2_ treated cultures presented multi-cell aggregates and residual populations through 15 days. A persistent yield of the cyanotoxin MC-LR (µg/cell) was observed in all ZnCl_2_ treated cells by 15 days, indicating that this cyanotoxin remains present in the environment even with low cell concentrations. This finding was supported by qPCR data gene expression profiles of *Mmt* and *mcyC*, suggesting that *M. aeruginosa* UTEX LB 2385 possesses several metal response mechanisms.

## 5. Materials and Methods 

### 5.1. Growth Monitoring

*Microcystis aeruginosa* UTEX LB 2385 (NCBI Taxon ID: 1296356) was acquired from UTEX Culture Collection of Algae, TX, USA, and was grown to late exponential growth phase (OD_750 nm_ = 1.0; cells/mL = 6 × 10^7^) in sterile 1X Cyanobacteria BG-11 Freshwater Medium (Sigma Life Sciences, St. Louis, MO, USA), within a sterile 250 mL Borosilicate Erlenmeyer flask at 21 ± 2 °C under constant 24 h cool-white fluorescent light (24 µmol⋅m^−2^ s^−1^ photons) at a constant agitation of 100 rpm (Innova 2000 Platform Shaker—New Brunswick Scientific, Edison, NJ, USA). The 1X Cyanobacteria BG-11 media was prepared with sterile deionized Milli-Q water (Milli-Q Plus ultra-pure water system—Millipore, Billerica, MA, USA) and the pH was adjusted to 8.0 ± 0.1 with 1 M NaOH. Duplicate sets of four 20 mL volumes of *M. aeruginosa* UTEX LB 2385 culture were centrifuged at 2900× *g* for 10 min, the supernatants were discarded, and the cell pellets were gently washed with sterile deionized Milli-Q water. These cells were repelleted and constituted in 20 mL fresh sterile 1X Cyanobacteria BG-11 media. The 20 mL volumes were aseptically transferred and diluted with 80 mL fresh sterile 1X BG-11 Medium into eight new sterile 250 mL Borosilicate Erlenmeyer Flasks.

### 5.2. Experimental Design

A sterile 1% zinc chloride (ZnCl_2_) (Sigma-Aldrich, St. Louis, MO, USA) solution was added to each *M. aeruginosa* UTEX LB 2385 culture to yield relative ZnCl_2_ concentrations: 0 mg/L, 0.1 mg/L, 0.25 mg/L, and 0.5 mg/L (0 µM, 0.734 µM, 1.835 µM, 3.669 µM, respectively), as described previously [50,70]. These ZnCl_2_ concentrations were targeted through multiple growth curve analysis from past experiments, with concentrations as high as 10 mg/L ZnCl_2_ (data not shown). The rationale was predicated on a previously studied cyanobacterium, *Synechococcus* sp. IU 625 with ZnCl_2_. All ZnCl_2_-treated cultures were maintained at the same culture growth parameters described above and were monitored by turbidity observation at optical density (O.D._750 nm_) with an UltraSpec III (Pharmacia LKB—Pfizer, New York, NY, USA) for a predetermined time course of Days: 0, 1, 5, 8, 12, and 15. All *M. aeruginosa* UTEX LB 2385 culture cell counts were performed via hemocytometer at 400× magnification using an Olympus BH2 BHS-312 Trinocular Microscope (Olympus Corp, Waltham, MA, USA), and all experiments were repeated in triplicates. The standard deviations of duplicate set means were used to generate the growth curves. 

### 5.3. Phase Contrast Microscopy

To determine the physiological effects of ZnCl_2_ concentration on *M. aeruginosa* UTEX LB 2385 cultures, cell cultures were collected and imaged. All ZnCl_2_-treated *M. aeruginosa* UTEX LB 2385 cultures were collected during the set, predetermined time points (Days: 0, 1, 5, 8, 12, and 15) and immediately imaged with a Zeiss AxioLab A1 phase contrast microscope coupled with an AxioCam MrC camera (Carl Zeiss, Oberkochen, Germany) at 1000× total magnification.

### 5.4. Flow Cytometry

Sample cell size for ZnCl_2_-treated *M. aeruginosa* UTEX LB 2385 cultures was measured (FCM) in 10,000 events per sample within a calibrated MACSQuant Analyzer 10 (Miltenyi Biotec, Auburn, CA, USA) containing 405 _nm_, 488 _nm_, and 638 _nm_ lasers, using the forward scatter setting (FSC-A) for a set short term and long term course period of 8 days (0, 0.25, 0.5, 1, 5, 8). For each time point, 1 mL of each cell treatment was aseptically transferred into a 1.5 mL microcentrifuge tubes, and the parameters were set to gently resuspend before 100 µL was measured in FCM. Different cyanobacteria populations may exhibit distinct patterns of size, complexity, and autofluorescence in regards to colony formation and to their phycobilisome complex. Furthermore, the light harvesting components and phycobilisome complex has been shown to be affected by heavy metals in concentration dependent kinetics [71,72]. Allophycocyanin and phycoerythrin fluorescent intensities for *M. aeruginosa* UTEX LB 2385 cells were measured in a calibrated MACSQuant Analyzer 10 (Miltenyi Biotec, Auburn, CA, USA) for the set time course period of 8 days (data not shown). All flow cytometry histograms and statistical analysis of measurements were generated via FlowJo software analysis (FlowJo, Ashland, OR, USA) to compare the effects of varying concentrations of ZnCl_2_ treatment within all cultures. 

### 5.5. Total RNA Isolation and cDNA Synthesis

The ZnCl_2_ treatment (0.5 mg/L) was found to inhibit *M. aeruginosa* UTEX LB 2385 cell number and decrease turbidity by Day 8. Therefore, total RNA (*M. aeruginosa* UTEX LB 2385—0, 0.1, and 0.25 mg/L ZnCl_2_ for Days: 1, 5, 12, and 15) was isolated and purified using a modified Ambion^®^ RiboPure™ Kit (Ambion, Austin, TX, USA) approach. Homogenization and sample disruption preparation was prepared by adding a volume of 48 mL of 100% EtOH to 60 mL of Wash Solution Concentrate. One mL TRI Reagent was then aseptically mixed with 250 µL of cultures within sterile 2 mL microcentrifuge tubes. The mixtures were homogenized (by vortexing), sonicated 15 times with 3 s pulses (20% power) using a Branson Sonifier Cell Disruptor 200 (Emerson Industrial, St. Louis, MO, USA), and incubated for 5 min at room temperature. Homogenates were centrifuged at 12,000× *g* for 10 min at 4 °C, and supernatants transferred to new sterile 2 mL microcentrifuge tubes. RNA extraction was performed by aseptically adding a volume of 200 µL CHCl_3_ solution to each sample, tightly capping and vortexing the sample tubes at 700× *g* for 15 s, and then incubating at room temperature for 5 min. Each sample was centrifuged at 12,000× *g* for 10 min at 4 °C before 400 µL of aqueous phase was aseptically transferred to sterile 2 mL microcentrifuge tubes. Then, 200 µL of 100% EtOH was aseptically added to the 400 µL of aqueous phase and immediately vortexed at 700× *g* for 5 s. The sample mixtures were aseptically transferred to a filter cartridge placed within a collection tube, capped, and centrifuged at 12,000× *g* for 30 s at room temperature. Each sample flow-through was discarded and the filter cartridge (with bound RNA) was replaced within the same collection tube. Next, 500 µL of wash solution was aseptically added to the filter cartridges, capped, and centrifuged at 12,000× *g* for 30 s at room temperature. Each sample flow-through was discarded and the filter cartridge was replaced within the same collection tube. The same wash solution step was repeated. Then, the filter cartridges were transferred to new sterile collection tubes and 100 µL elution buffer was added to each respective filter column. The samples were then incubated at RT for 2 min and centrifuged at 12,000× *g* for 30 s to elute RNA.

The concentration (µg/mL) and A260/280 ratios for total RNA samples were checked using a BioDrop UV/VIS Spectrophotometer (Denville Scientific, Metuchen, NJ, USA). The isolated RNA samples served as templates for cDNA synthesis with an ABI High Capacity cDNA Reverse Transcription Kit (Applied Biosystems-Life Technologies, Camarillo, CA, USA), using random hexamers as per the manufacturer’s specification. Briefly for each sample, 10 µL DNase-treated RNA was carefully mixed with 10 µL 2X RT MasterMix I (2 µL RT Buffer; 0.8 µL 25X dNTP Mix; 2 µL 10X RT Random Primers; 1 µL MultiScribe™ Reverse Transcriptase; 4.2 µL sterile nuclease-free H_2_O) within a 200 µL nuclease-free reaction tube. The sample tubes were placed and run in a Veriti 96 well Thermocycler (Applied Biosystems, Camarillo, CA, USA) via incubation at 25 °C for ten minutes, 37 °C for two hours, and RT inactivation at 85 °C for 5 m. cDNA sample concentrations and A260/280 ratios were checked using a BioDrop UV/VIS Spectrophotometer, and stored at −20 °C until prepared to use. 

*Microcystis aeruginosa Mmt*, *mcyC,* and *mcyE* genes were chosen for primer design based upon phylogenetic analysis of 16S-23S rRNA ITS sequences, multiple alignment of *Microcystis aeruginosa* MC synthetase sequences, and multiple alignment of selected *Microcystis* MT amino acid sequences (T-Coffee Program) (data not shown). 

### 5.6. Quantitative Polymerase Chain Reaction (qPCR)

Oligonucleotides for qPCR were designed using IDT qPCR PrimerQuest (IDT, IA, USA) based on *Microcystis aeruginosa mt* gene (*M. aeruginosa* PCC 7941 (NZ_HE973171), and qPCR *mcyC* and *mcyE* oligonucleotides were designed based on *M. aeruginosa mcy* genes (*M. aeruginosa* UTEX LB 2388: EU009881 and *M. aeruginosa* K-139: AB032549, respectively) using IDT qPCR PrimerQuest. The following primer pairs were generated: MmtRT2f: 5ʹ-TTGTGAATCCTGTACGTGTCAA-3ʹ and MmtRT2r: 5ʹ-GTCCACAGCCTTTCCCTTTA-3ʹ; mcyC_RT1f: 5ʹ-GGCTAAACCTGACGGGTATAAA-3ʹ and mcyC_RT1r: 5ʹ-CGCAATATTGAGGGAACACAAG-3ʹ; mcyE_RT1f: 5ʹ-AAGTGGGACCAAGACCAATAC-3ʹ and mcyE_RT1r: 5ʹ-TCTAAGCCACGATTGAGAGAAC-3ʹ. An endogenous control was designed to normalize the relative gene expression of *mcyC*, *mcyE*, and *mt* genes using IDT PrimerQuest with *M. aeruginosa* UTEX LB 2385 (KF372572) 16S ribosomal RNA {MA8516S_RT1F: 5ʹ-GTAGCAGGAATTCCCAGTGTAG-3ʹ and MA8516S_RT1R: 5ʹ-TTCGTCCCTGAGTGTCAGATA-3ʹ}.

The cDNA samples were diluted to a concentration of ~100 µg/mL and measured with a BioDrop UV/VIS Spectrophotometer to determine A260/280 ratios. The comparative *C*_T_ relative gene expression method was used with the final equation:

Fold change = 2^−ΔΔ*C*t^ (1)

Quantitative RT PCR reactions were performed within 96-well plate assays using an Applied Biosystems StepOnePlus™ Real Time PCR System (ThermoFisher Scientific, MA, USA) with a Luna^®^ Universal qPCR Master Mix (New England BioLabs, Ipswich, MA, USA) (containing Hot Start *Taq* DNA Polymerase). SYBR green dye chemistry was used for reactions containing: 10 µL qPCR Master Mix, 7 µL nuclease-free H_2_O, 1 µL of forward and 1 µL reverse primers for a total final 1 µM concentration, and 1 µL < 100 ng final mass of diluted cDNA. The qPCR reactions were performed in triplicates through an initial incubation of 50 °C for two minutes, an initial one-cycle denaturation step at 95 °C for 60 s, and 40 cycles of 95 °C denaturation for 15 s with 60 °C extension for 30 s. 

### 5.7. Enzyme-Linked Immunosorbent Assay (ELISA)

ZnCl_2_-treated *M. aeruginosa* UTEX LB 2385 cultures were collected for a predetermined course of 15 days (Days: 1, 5, 8, 12, and 15) by centrifuging at 2900× *g* for 10 min at RT. The supernatants were aseptically removed from the pellets and filtered into new sterile centrifuge tubes using sterile microfiltration apparatus (with sterile ≤ 0.22 µm membrane filters). All pellet and supernatant samples were frozen at −20 °C and stored for 15 days. Intracellular microcystin samples were extracted via modified extraction methods [22,73]. Briefly, frozen *M. aeruginosa* UTEX LB 2385 pellet samples were thawed, refrozen at −20 °C and thawed again, before 5 mL 75% methanol was added and samples were sonicated for 1 min at 20% power using a Branson Sonifier Cell Disruptor 200 (Emerson Industrial, St. Louis, MO, USA). The homogenized pellet samples were allowed to sit at RT for 5 min before they were transferred to a sterile microfiltration device/apparatus (with a sterile ≤ 0.22 µm membrane filter). For initial supernatant samples, 5 mL 75% methanol was added and mixed, before aseptically transferring to their respective sterile microfiltration device/apparatus. The total filtrates were diluted to final 1 mL 4% methanol working sample solutions.

ELISA quantitation for intracellular and extracellular microcystin-LR equivalents was performed using a Microcystin-LR ELISA kit (colorimetric) (Abnova, Taipei, Taiwan) as per manufacturer specifications. Final absorbances were read at 450 nm in duplicates using a Varioskan™ LUX multimode microplate reader (ThermoFisher Scientific, Waltham, MA, USA) with SkanIt Software. 

### 5.8. Statistical Analysis

The growth analysis experiments were performed in triplicates and the standard deviation of means were used to generate growth curves. The statistical analysis of flow cytometry measurements (FCM) were generated via FlowJo software analysis (FlowJo, Ashland, OR, USA). The initial raw data was analyzed using the comparative C*_t_* method in the ABI StepOne Software (Life Technologies, Camarillo, CA, USA). To ascertain if the endogenous 16S rRNA reference gene varied due to experimental conditions, student’s *t*-tests were performed for all accumulated averaged *C*_t_ values organized as treatment groups [74]. The full form of the comparative C*_t_* method equation (1) was used to evaluate relative quantifications of *mcyE*, *mcyC*, and *mt* genes using the MA_UTEX LB 2385-specific 16S rRNA endogenous control for the calculations. A Welch two sample t-test was used to evaluate possible statistical significance between different, pooled ZnCl_2_ treatment groupings of 16S rRNA endogenous control, using RStudio statistical software (RStudio Team, 2018). A standard 2^nd^-order polynomial equation (y = 7.5536x^2^ −54.927x + 96.06) was used to calculate MC-LR equivalents below the 2.5 µg/L standard range. Total MC-LR (µg/cell) quota was calculated by adding intracellular-extracellular MC-LR equivalents (µg/L) and dividing by average cells/L. A Pearson’s product-moment *r* correlation was used to evaluate increasing ZnCl_2_ concentration.
